# A Case of Persistent Muscle Cramps in an American Football Player With Cystic Fibrosis

**DOI:** 10.7759/cureus.8621

**Published:** 2020-06-14

**Authors:** Patrick Wise, Lindsay Lafferty, Shawn F Phillips

**Affiliations:** 1 Family and Community Medicine, Penn State College of Medicine, Hershey, USA; 2 Family and Community Medicine/Orthopedics and Rehabilitation, Penn State Health Milton S. Hershey Medical Center, Hershey, USA; 3 Family and Community Medicine/Orthopedics and Rehabilitation, Penn State Health/College of Medicine, Hershey, USA

**Keywords:** cystic fibrosis, muscle cramps, hyponatremia, sports medicine, sports nutrition

## Abstract

Exercised-associated muscle cramp (EAMC) is a common occurrence in sports medicine. We highlight a 17-year-old male high-school football player with a history of cystic fibrosis and hyponatremic seizure, who presented for recurrent EAMC. After establishing an appropriate electrolyte replacement and hydration plan, he was able to complete his season with minimal symptoms. This case highlights the importance of hydration and nutrition planning when managing athletes with cystic fibrosis.

## Introduction

Exercise-associated muscle cramp (EAMC) is a common condition among athletes [1-3]. Cystic fibrosis (CF) is an autosomal recessive disorder involving malfunctioning chloride channels. As treatments for CF have improved, it is likely that more individuals with CF will become active in sport. Athletes with CF can have significant electrolyte disturbances and associated complications [4-6]. It will be important for physicians and other sports medicine professionals to be aware of potential medical complications for athletes with CF [7]. Here, we report a case of EAMC and hyponatremia in a football player with CF. This case was presented at the 2018 American Medical Society of Sports Medicine National Conference in Orlando, FL. Ahead of that conference, the abstract for this case was published with other presented cases in the March 2018 edition of the Clinical Journal of Sports Medicine [8].

## Case presentation

A 17-year-old male high-school football offensive and defensive lineman with a history of CF presented with recurrent muscle cramping during football. He follows with the CF clinic, and his pulmonary function has been excellent. Over the course of two football seasons, he consistently experienced severe muscle cramps, which would last 30 minutes to three hours. These cramps would usually occur during second half of games and two-a-day workouts. The cramps affect mainly his lower extremities, but also occasionally in the abdomen and upper extremities. His primary strategy to treat and prevent cramps was hydrating with water. Two seasons prior to presenting in sports medicine clinic, he experienced a hyponatremic seizure, with a serum sodium of 121 mmol/L (normal range 136-146 mmol/L). This occurred after the second practice session of the day. He was seen in follow-up by the CF clinic and referred to a nutritionist, who made recommendations on electrolyte replacement. Based on this advice, he started consuming multiple salt tabs “throughout the day” on game days and hydrating with two to three bottles of a commercially available sport drink and one to two bottles of a pediatric electrolyte replacement solution during his football games. Despite these modifications, he continued to experience severe cramping during the second half of football games, and was unable to finish most games. He was referred to sports medicine for further evaluation and treatment recommendations.

During initial assessment, his clinical examination was generally unremarkable, but was notable for digital clubbing. Initial laboratory testing showed a sodium of 140 mmol/L and a potassium of 4.3 mmol/L (normal 3.5-5.1 mmol/L). He was instructed to document his weight before and after practices and games. He was restricted from practice if he did not achieve his pre-practice weight prior to the next practice. He averaged three pounds of weight loss during practice and seven pounds of weight loss during his first game. In game hydration was restricted to an adult rehydration solution, which contains 45 mEq sodium, 20 mEq potassium, 35 mEq chloride, and 16 g dextrose. His own personal bottle of electrolyte solution was brought to him even during timeouts. He averaged drinking 32 ounces of this electrolyte solution per half (64 ounces per game). He ingested one 1,000 mg salt tablet (43 mEq of sodium) at the beginning of each game and again at half time. With this regimen he successfully played and finished every game during the 2017 season as a two-way starter on both the offensive and defensive lines. He averaged four pounds of weight loss during games. While, due to cost and availability, it was not possible to check his serum sodium level around practices, his serum sodium level was checked after his first game of 2017. At this time, his post-game serum sodium level was 131 mmol/L. After this measurement, he was instructed to replete sodium immediately post-practice and games. His post-game salty meal usually consisted of a cup of ramen noodles, which average about 1,700 mg of sodium. He had two short episodes of calf cramping during the season, but was able to return to play in the same game. He is considering playing next year at the collegiate level and will follow up in sports clinic prior to starting college. 

## Discussion

EAMC is defined as painful involuntary spasmodic contraction of skeletal muscle occurring during or immediately after exercise [1]. The etiology of EAMC remains unclear. EAMC-prone athletes, and specifically EAMC-prone American football players, have been shown to exhibit decreased blood sodium and potassium levels compared to controls. These decreased levels have are thought to be a result of either increased physiological electrolyte loss or decreased electrolyte intake during sport participation [2,3].

There is a paucity of literature on athletes with CF, specifically in the sport of football. Athletes with CF are at increased risk for electrolyte imbalances due to underlying physiology. The cystic fibrosis transmembrane regulator (CFTR) is an active chloride channel that is found within epithelial cells of many organs, including the skin. In sweat glands, defective CFTR results in decreased reabsorption of sodium chloride within the duct and therefore saltier sweat. This is the basis for the diagnostic testing for CF, and the physiology behind electrolyte disturbances for athletes with CF. The excessive sodium chloride loss experienced can lead to hyponatremia, hypochloremia, excess water loss, and extracellular fluid (ECF) contraction [4]. Ultimately, secondary hyperaldosteronism can develop and potassium wasting occurs through excess potassium secretion in sweat and urine [5].

For athletes with CF, rehydration with hypotonic solution can exacerbate exertional hyponatremia. To properly maintain volume and electrolyte levels, athletes with CF require maintenance of both volume and electrolyte concentration. It is imperative to ingest appropriate electrolytes and glucose during and surrounding training and competition. Sodium ingested is absorbed into the small intestine apically through epithelial sodium channel (ENaC) and sodium-glucose co-transporter 1 (SGLT1). Once apically absorbed, sodium is then absorbed basolaterally into the bloodstream through Na^+^/K^+^-ATPase and potassium is reabsorbed through a specific potassium channel. An electrochemical gradient created from the sodium transport facilitates passive chloride transport and water is able to then follow [6].

For this athlete, the salt ingestion was integral in maintaining sodium levels during activity. Therefore, salt loading was advised immediately before games, at halftime, and immediately post. To facilitate the co-transport of sodium, we encouraged him to take in additional glucose either with a small sugary snack, such as a handful of jelly beans, or his adult rehydration solution. Hydration, restricted to adult electrolyte solution, was a mainstay of his regimen. He was encouraged to drink as much rehydration solution as tolerable before, during, and after participation. It should be noted that gastrointestinal discomfort is a limiting factor in ingestion of this amount of salt and rehydration solution. Ingestion of salt tabs, snacks, and adult rehydration solution was extremely effective for this athlete. The nutritional strategy applied for this athlete allowed him to participate with few symptoms and no complications throughout the season.

Exercise has been shown to improve quality of life in people living with CF, but can be complicated by electrolyte disturbances [7]. Relatively simple alteration of hydration practices was employed to allow this athlete to continue with high-level exercise and athletic competition. 

## Conclusions

Participation in sport is an important way that many young people stay physically active. As the available treatments for CF continue to improve, sports medicine physicians and athletic trainers will encounter more individuals with CF participating in athletics. This article reviewed the case of an athlete who presented with an extreme presentation of a common condition, EAMC. His pronounced presentation was precipitated by an underlying diagnosis of CF. In order to properly care for athletes with CF, it will be important for sports medicine providers to understand the physiology and the associated electrolyte loss seen in CF and to keep these physiological differences in mind when treating otherwise common conditions. 
